# Radical Cure for Piles

**Published:** 1876-04

**Authors:** 


Radical Cure for Piles.—Dr. A. B. Bowen, of Magnoketa, Iowa, writes: "In a recent number of 'The Record' my attention was directed to the treatment for nævus by hypodermic injection. From the similarity of the anatomical structure of nævus to hæmorrhoidal tumors, I was induced to try the remedy. In the latter I used carbolic acid and ergot (fl. ext.) in equal parts, injecting from ten to fifteen minims of the solution into the spongy, vascular hæmorrhoidal tumor. This was repeated about once a week for five or six times, when the tumor had entirely disappeared. I have tried this in several cases, and it acts like a specific."—*American Medical Weekly.*

